# Is the Arterial Anastomosis Time a Key Predictor of Free Flap Failure in Oncological Head and Neck Reconstruction? A Prospective Cohort Study

**DOI:** 10.3390/jcm15176819

**Published:** 2026-09-02

**Authors:** Cristina Cárdenas Serres, Fernando Almeida Parra, Álvaro Ranz Colio, Ángela Bueno de Vicente, Patricia de Leyva Moreno, Verónica de Prado Mendoza, Jorge Nuñez Paredes, Manuel Picón Molina, Julio Acero Sanz

**Affiliations:** 1 Department of Oral and Maxillofacial Surgery, Ramón y Cajal University Hospital, 28034 Madrid, Spain; 2 Ramón y Cajal Institute of Sanitary Research (IRYCIS), 28034 Madrid, Spain

**Keywords:** free flap, microsurgery, head and neck reconstruction, arterial anastomosis, flap failure, vasospasm, ischaemia time, oncological reconstruction, prospective study

## Abstract

**Background/Objectives:** Free flap microsurgical reconstruction is the gold standard for complex oncological head and neck defects. Despite high success rates, flap failure carries significant morbidity and oncological implications. This study aimed to identify preoperative, analytical, and intraoperative predictors of free flap non-viability in a prospective series of head and neck oncological reconstructions. **Methods:** A prospective observational cohort study included 98 consecutive patients undergoing microsurgical free flap reconstruction at a single tertiary centre (January 2022–December 2025). Categorical variables were analysed with Fisher’s exact test and odds ratios (OR) with 95% confidence intervals (CI); continuous variables were analysed using the Mann–Whitney U test and rank-biserial correlation (r). Multivariate logistic regression was performed for variables with *p* < 0.20 in univariate analysis. **Results:** Free flap survival was 88.8% (87/98). On univariate analysis, intraoperative vasospasm (9.2% vs. 72.7%; *p* < 0.001) and prolonged arterial anastomosis time (median 25 vs. 45 min; *p* = 0.012) showed the strongest associations with flap failure. A negative O’Brien patency test (*p* = 0.012) and greater number of transfused red blood cell units (*p* = 0.033) were also significant. No preoperative variable was significantly associated with flap outcome. On multivariate analysis, vasospasm remained the sole independent predictor of viability (*p* = 0.014), with arterial anastomosis time showing a persistent trend (*p* = 0.079). **Conclusions:** Intraoperative vasospasm was the principal independent determinant of flap viability, while prolonged arterial anastomosis time showed a consistent univariate association and a non-significant multivariable trend. Given the small number of flap failures (n = 11), these findings, together with the proposed intraoperative time thresholds, should be regarded as preliminary and hypothesis-generating pending external validation. These findings underscore the primacy of intraoperative technical and vascular factors and may guide surgical training and perioperative management protocols.

## 1. Introduction

Free flap microsurgical reconstruction represents the cornerstone of surgical management for complex oncological defects of the head and neck, enabling reconstruction of extensive bone and soft tissue defects following ablative oncological surgery [1]. As reflected by large prospective and retrospective series, success rates have progressively reached 91–99% in specialised centres [2,3,4], owing to technical refinements in anastomotic technique, standardisation of postoperative monitoring protocols, and advances in anaesthetic management.

Despite this high reliability, free flap failure remains a clinically significant complication. Flap loss necessitates additional surgical intervention, prolongs hospitalisation, delays adjuvant oncological treatment, and may result in permanent functional and aesthetic sequelae [5,6]. Identification of modifiable risk factors is therefore a research priority, both to guide intraoperative decision-making and to improve patient selection and perioperative planning.

The determinants of free flap failure can be broadly categorised into patient-related factors (age, comorbidities, nutritional status, hypercoagulable states), disease-related factors (tumour characteristics, prior radiotherapy, previous flap failure), and intraoperative technical and haemodynamic factors (anastomosis quality and time, ischaemia duration, vasospasm, haemodynamic instability) [1,6]. Among these, the relative contribution of each category remains a matter of ongoing investigation, particularly the role of preoperative coagulopathy versus intraoperative technical factors.

The coagulation status of oncological patients presents particular concerns in the microsurgical setting. Cancer-associated hypercoagulability, characterised by increased levels of fibrinogen, activated coagulation factors, and reduced natural anticoagulants, may predispose to anastomotic thrombosis and flap loss [7,8]. Multiple thrombophilic conditions—including Factor V Leiden mutation, Prothrombin G20210A variant, antiphospholipid antibody syndrome, protein C and S deficiencies, and thrombocytosis—have been implicated in individual case reports and small series as risk factors for microvascular failure [9,10,11,12,13]. However, whether systematic preoperative thrombophilia screening meaningfully predicts free flap outcome in the oncological context remains unresolved [7,8,14].

Ischaemia time—defined as the interval between donor vessel ligation and completion of reperfusion—is a widely debated intraoperative variable. Experimental evidence demonstrates that even brief ischaemia induces progressive metabolic acidosis, electrolyte imbalance, and activation of the coagulation cascade within the flap tissue [15]. In clinical series, the relationship between ischaemia duration and complications has been inconsistent: some studies have demonstrated a significant association between prolonged ischaemia and flap complications [16,17,18,19], while others—particularly in head and neck cohorts—have not confirmed this relationship [20]. This heterogeneity may reflect differences in flap type, ischaemia measurement methodology, and confounding by technical factors.

Intraoperative vasospasm is a well-recognised cause of reduced flap perfusion and anastomotic thrombosis. Its management requires rapid recognition and targeted pharmacological intervention, yet current evidence regarding optimal vasodilatory agents remains limited and largely derived from experimental models [21,22]. The interaction between vasospasm and anastomosis time—both mechanistically linked and clinically intertwined—has been rarely investigated in prospective cohorts.

The present prospective cohort study was designed to systematically investigate these poorly defined determinants by simultaneously evaluating epidemiological, analytical, coagulation, and intraoperative variables as potential predictors of free flap viability in a consecutive series of oncological head and neck reconstructions performed at a single tertiary institution between January 2022 and December 2025. Special attention was given to arterial anastomosis time as a novel and potentially modifiable intraoperative determinant of flap outcome.

## 2. Materials and Methods

### 2.1. Study Design and Setting

A prospective observational cohort study was conducted at a single tertiary-level academic hospital with a dedicated Head and Neck Oncology and Reconstructive Microsurgery Unit. The study period spanned from January 2022 to December 2025, encompassing 98 consecutive adult patients who underwent free flap microsurgical reconstruction of the head and neck. The study was conducted in accordance with the Declaration of Helsinki and received approval from the institutional Ethics Committee. Written informed consent was obtained from all participants.

### 2.2. Inclusion and Exclusion Criteria

Patients were eligible if they were aged ≥18 years and underwent microsurgical free flap reconstruction of the head and neck at our institution during the study period. Patients were excluded if they had incomplete preoperative analytical data, or if the surgical procedure was aborted prior to anastomosis completion. All procedures were performed by consultant surgeons with adequate level microsurgical training.

### 2.3. Variables Collected

Data were prospectively recorded for each patient by dedicated research personnel and encompassed: (i) epidemiological variables (age, sex, smoking, alcohol consumption); (ii) preoperative comorbidities (hypertension, dyslipidaemia, diabetes mellitus, ischaemic heart disease, peripheral arterial disease, chronic liver and kidney disease, BMI > 30 kg/m^2^, personal and family history of venous thromboembolism, reduced mobility, active oncological process); (iii) pharmacological history (anticoagulants, antiplatelet agents); (iv) preoperative analytical parameters, including full blood count, renal and hepatic function markers, coagulation profile (fibrinogen, prothrombin activity, aPTT, prothrombin time, D-dimer, protein C, protein S, antithrombin), and thrombophilia markers (Factor V Leiden, Prothrombin G20210A, anticardiolipin antibodies IgG/IgM, anti-β2-glycoprotein I antibodies IgM/IgG); (v) thromboembolic risk scores (Padua Prediction Score; IMPROVE Score); (vi) surgical variables including flap type, reconstruction timing, prior microsurgical history, prior radiotherapy, recipient vessels, surgery and anastomosis durations, ischaemia time, O’Brien patency test, intraoperative critical vasospasm (defined as vasospasm with complete arrest of arterial blood flow requiring pharmacological or mechanical intervention), mean arterial pressure (MAP) episodes < 75 mmHg, red blood cell transfusion requirements, and vasopressor use.

The primary outcome was free flap viability at hospital discharge, defined as complete survival of the reconstructed unit. Non-viability was defined as complete surgical removal due to irreversible vascular compromise at any point during the inpatient period.

### 2.4. Statistical Analysis

Continuous variables are expressed as median with interquartile range [IQR]. Categorical variables are reported as frequencies with 95% Wilson confidence intervals. For inferential analysis between viable and non-viable groups, Fisher’s exact test was applied to categorical variables with calculation of OR and 95% CI. The Mann–Whitney U test was used for continuous variables, with the rank-biserial correlation coefficient (r) reported as effect size. Univariate logistic regression was performed for all variables, and those with *p* < 0.20 were entered into a multivariate logistic regression model with mild L2 regularisation (λ = 0.1) to mitigate overfitting. Wald tests were used for *p*-values and confidence intervals in regression models. All analyses were performed with Python 3.12. Two-sided *p* < 0.05 was considered statistically significant.

## 3. Results

### 3.1. Patient Characteristics

A total of 98 consecutive patients were included. Median age was 64 years (IQR 54–74; mean 62.9 ± 14.4) and 56 (57.1%) were male. Baseline characteristics are detailed in Table 1. The most prevalent comorbidities were hypertension (43.9%), dyslipidaemia (29.6%), and diabetes mellitus (12.2%). Active oncological disease was present in 71 patients (72.4%). Of 81 patients with documented histology, squamous cell carcinoma was most frequent (71.6%), followed by adenoid cystic carcinoma (6.2%) and ameloblastoma (6.2%). Prior radiotherapy to the operative field had been received by 24 patients (24.5%), twenty-eight patients (28.6%) had a history of prior head and neck surgery, of whom 14 (14.3%) had undergone a previous microsurgical reconstruction and 11 (11.2%) had a history of flap failure.

Anticoagulant therapy was documented in 11 patients (11.2%), principally rivaroxaban (n = 4), acenocoumarol (n = 4), and low-molecular-weight heparin (n = 1). Of the 7 patients with a personal history of venous thromboembolism, the documented events included deep vein thrombosis associated with a peripherally inserted central catheter (n = 1), popliteal vein thrombosis (n = 1), jugular and sphenoid sinus thrombosis (n = 1), and pulmonary embolism (n = 1).

### 3.2. Surgical Characteristics

The most commonly performed flap was the fibula osteomyocutaneous flap (n = 30; 30.6%), followed by radial forearm (n = 24; 24.5%), anterolateral thigh (n = 18; 18.4%), and vastus lateralis flaps (n = 14; 14.3%). Primary reconstruction was performed in 82 cases (83.7%). The facial artery was the most frequently used arterial recipient (n = 66; 67.3%), followed by the thyroid artery (n = 10; 10.2%), temporal artery (n = 7; 7.1%), lingual artery (n = 7; 7.1%), and external carotid artery (n = 6; 6.1%). Venous anastomosis was performed most commonly to the thyrolingual trunk (n = 42; 42.9%), facial vein (n = 32; 32.7%), and external jugular vein (n = 8; 8.2%).

Median surgery duration was 470 min [390–602]. Median total ischaemia time was 55.5 min [35–95], exceeding 90 min in 29 patients (29.6%). Median arterial and venous anastomosis times were 25 min [19–35] and 35 min [29–44], respectively. A positive O’Brien patency test was recorded in 83 patients (84.7%). Intraoperative vasospasm occurred in 16 patients (16.3%). Red blood cell transfusion within 24 h was required in 70 patients (71.4%), and vasopressors were used in 66 (67.3%). Median total time with MAP < 75 mmHg in the first 24 h was 108 min [75–318], representing a median 7.5% of the monitoring period. Surgical data are shown in Table 2.

### 3.3. Free Flap Viability and Failure Analysis

Of 98 patients, 87 flaps (88.8%) were viable at discharge and 11 (11.2%) required surgical removal due to irreversible vascular compromise. Among failed flaps, the predominant mechanism was arterial failure or ischaemia—due to arterial thrombosis or vasospasm—in 8 cases (72.7%), while venous congestion secondary to venous thrombosis accounted for the remaining 3 cases (27.3%).

When stratified by flap category, non-viability was more frequent among osseous flaps (6/39; 15.4%) than among soft tissue flaps (5/59; 8.5%). Within the osseous group, failures occurred in the fibula osteomyocutaneous flap (4/30; 13.3%), the iliac crest osteomuscular flap (1/6; 16.7%), and the dorsal scapular chimeric flap (1/3; 33.3%). Within the soft tissue group, losses were recorded in the radial forearm fasciocutaneous flap (3/24; 12.5%) and the anterolateral thigh fasciocutaneous flap (2/18; 11.1%), while neither the vastus lateralis myocutaneous flap (0/14) nor the transverse rectus abdominis myocutaneous flap (0/3) experienced any failure.

Twenty-five of 98 patients (25.5%) required surgical re-intervention during the index admission. Ten underwent active surgical revision of the microanastomosis due to vascular compromise: four for arterial thrombosis or ischaemia, of whom one flap was salvaged (25.0%), and six for venous congestion or thrombosis, of whom five flaps were successfully rescued (83.3%). Overall, anastomotic salvage was achieved in 6 of these 10 cases (60.0%). A further seven patients required surgical re-exploration for direct flap removal due to irreversible microanastomosis failure; all seven resulted in flap loss. The remaining eight re-interventions were attributable to non-vascular complications and included wound dehiscence (n = 4), cervical haemorrhage requiring surgical evacuation (n = 2), emergency tracheostomy (n = 1), and foreign body removal (n = 1); all eight flaps in this subgroup survived to discharge.

### 3.4. Comparative Analysis

#### 3.4.1. Epidemiological and Comorbidity Variables

No statistically significant difference was identified between viable and non-viable groups for any epidemiological variable, comorbidity, pharmacological history, or oncological characteristic (Table 3). This included prior radiotherapy (*p* = 1.000), previous flap failure (*p* = 0.106), anticoagulant use (*p* = 0.607), and active oncological process (*p* = 1.000).

#### 3.4.2. Preoperative Analytical and Coagulation Variables

Preoperative analytical variables showed no clinically meaningful differences between groups (Table 4). Serum creatinine was the only parameter reaching statistical significance, being lower in the non-viable group (median 0.65 vs. 0.88 mg/dL; *p* = 0.009). This is interpreted as an incidental finding, most likely reflecting differences in body composition rather than a causal biological relationship. Fibrinogen (*p* = 0.317), prothrombin activity (*p* = 0.151), aPTT (*p* = 0.392), D-dimer (*p* = 0.585), protein C (*p* = 0.520), protein S (*p* = 0.248), and antithrombin (*p* = 0.063) were all non-significant. When these coagulation parameters were re-examined as continuous variables in a complementary analysis, none reached statistical significance, confirming the robustness of these null findings. Thrombophilia genotyping (Factor V Leiden heterozygous 2.0%; Prothrombin G20210A heterozygous 4.1%) and antiphospholipid antibodies showed no significant association with flap outcome. Neither the Padua score (*p* = 0.813) nor the IMPROVE score (*p* = 0.122) differed significantly.

#### 3.4.3. Intraoperative Variables

**Figure 1:**
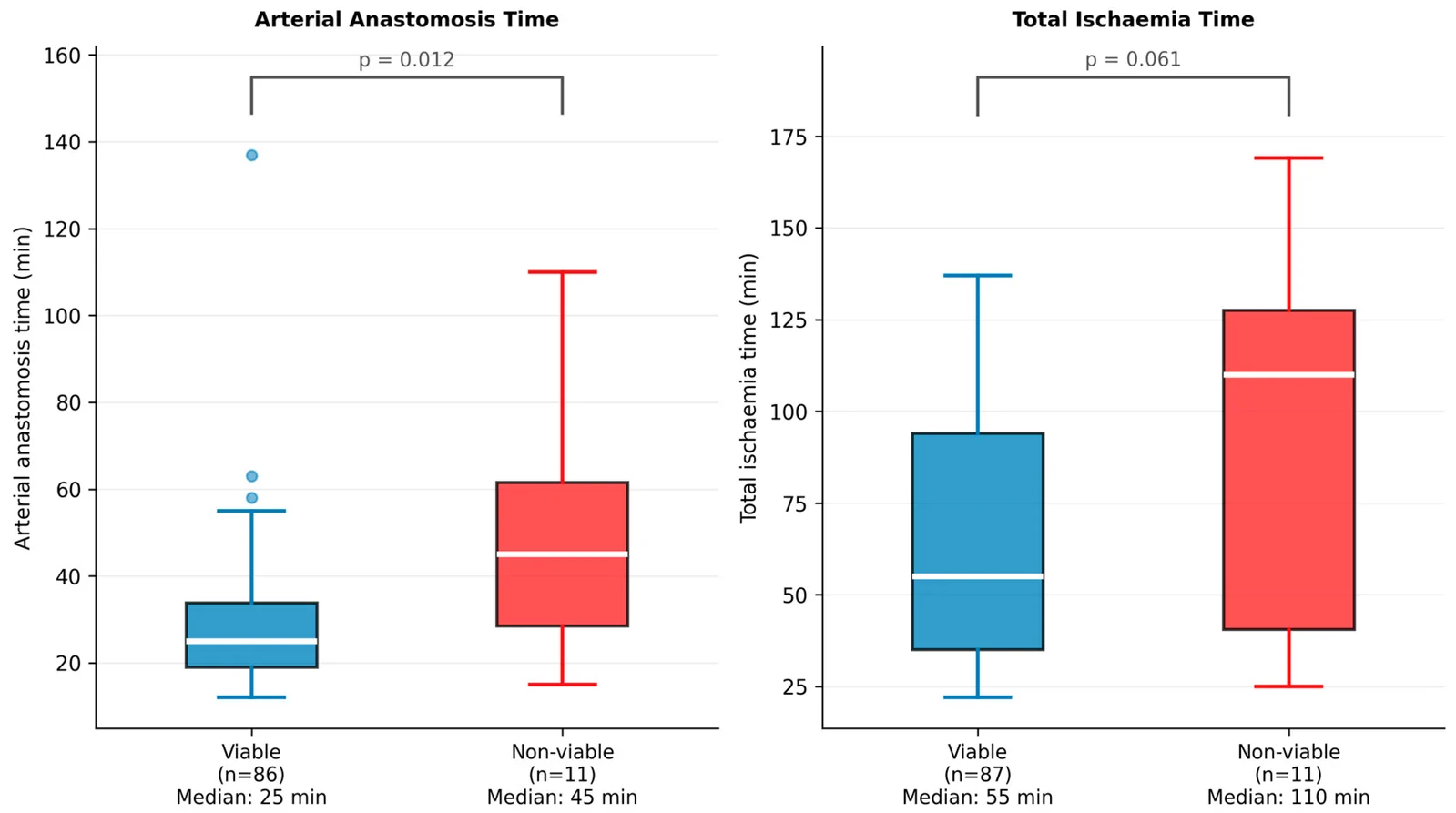
Intraoperative time variables by free flap viability outcome. Box plots of arterial anastomosis time ((**left**); Mann–Whitney U *p* = 0.012) and total ischaemia time ((**right**); *p* = 0.061) in viable (blue) and non-viable (red) flaps. Whiskers show 1.5 × IQR; individual outliers shown as points.

**Figure 2:**
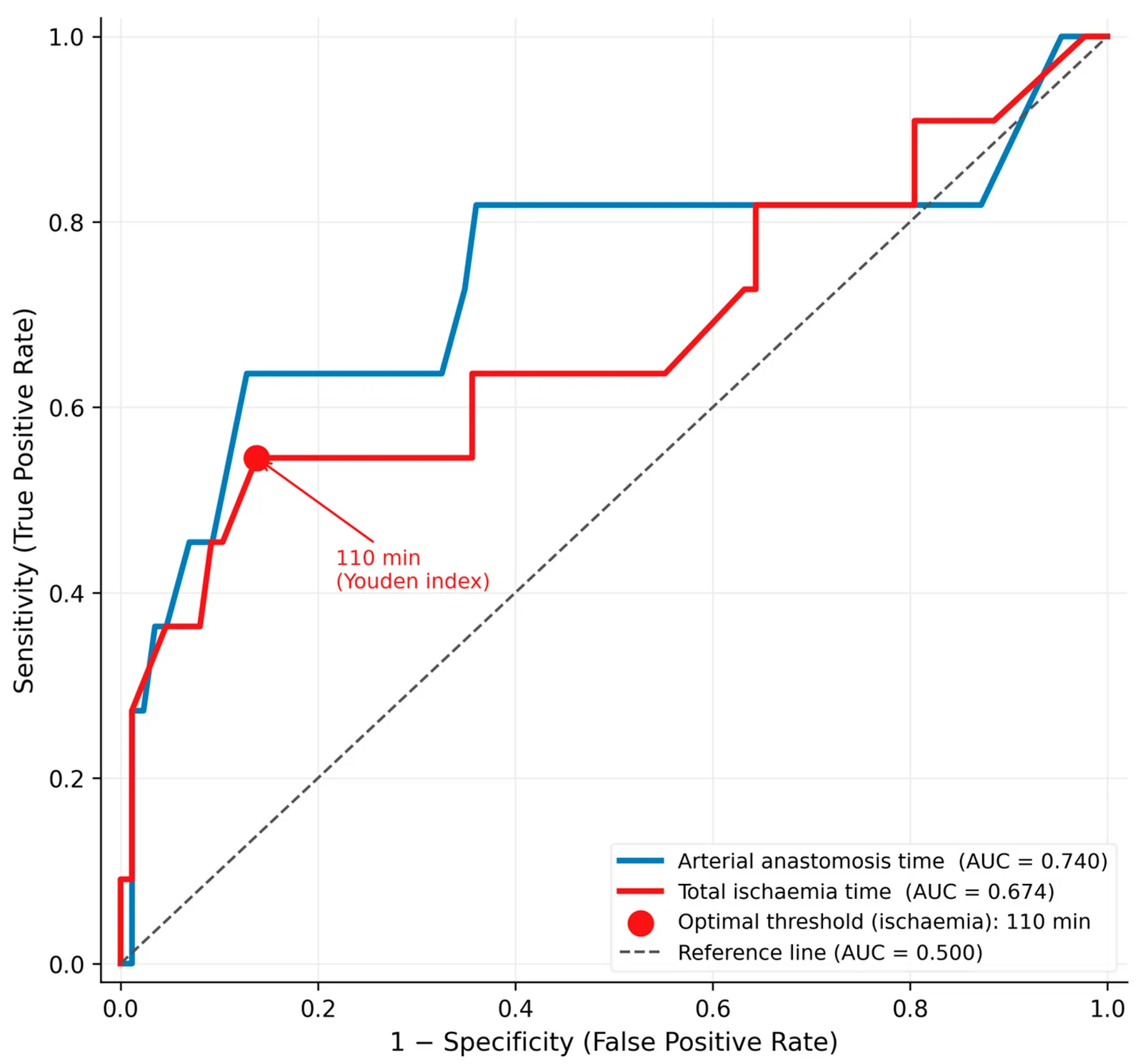
ROC curves for continuous predictors of free flap non-viability. Receiver operating characteristic curves for arterial anastomosis time (AUC = 0.740, blue) and total ischaemia time (AUC = 0.674, red). The optimal Youden threshold for ischaemia time (110 min) is indicated. Reference line (AUC = 0.500) shown as dashed diagonal.

**Figure 3:**
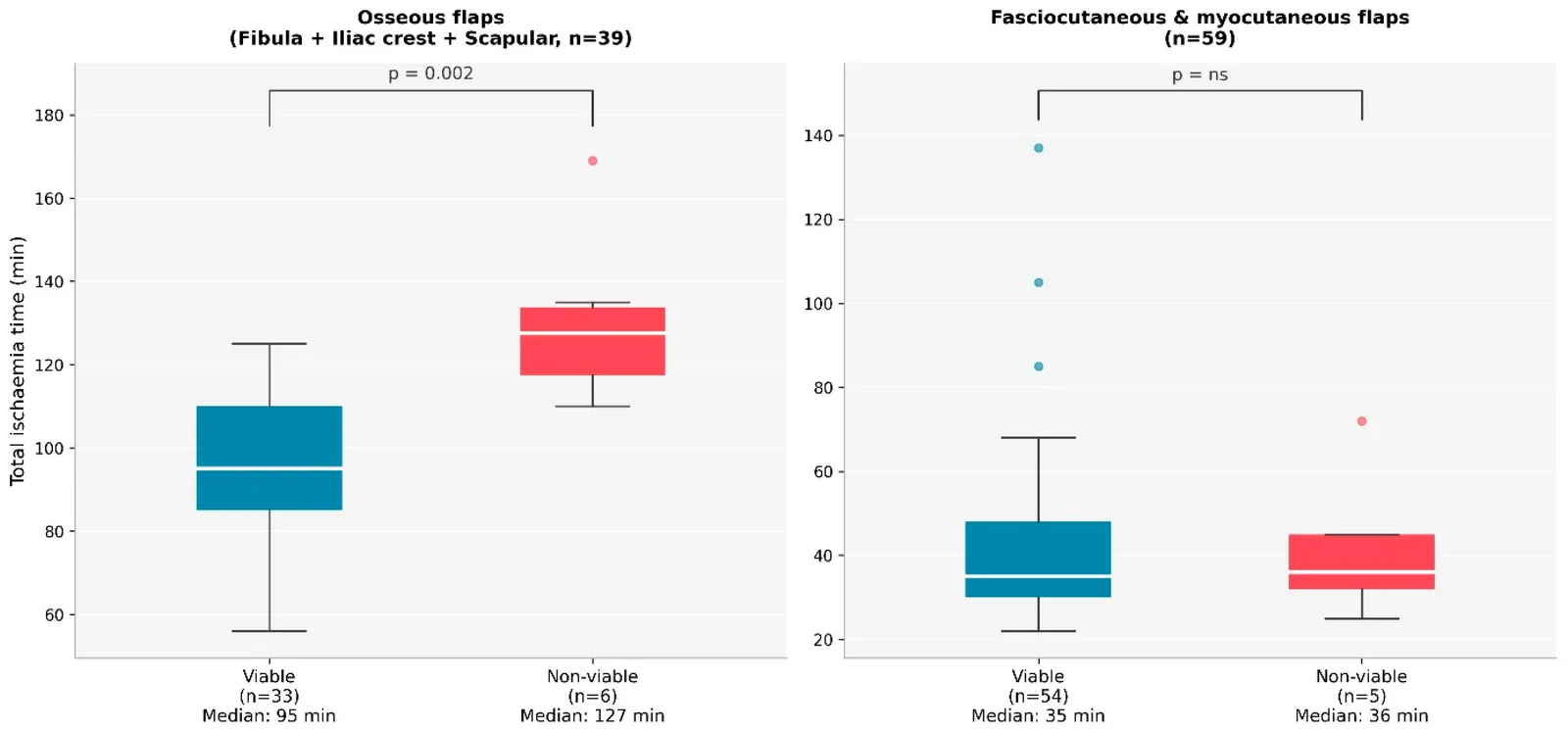
Ischaemia time by flap category and viability outcome. Total ischaemia time in viable (blue) and non-viable (red) flaps, stratified by osseous flaps (fibula + iliac crest + scapular, n = 39; *p* = 0.002) and fasciocutaneous/myocutaneous flaps (n = 59; *p* = ns). The ischaemia–failure association was driven exclusively by osseous flaps.

Intraoperative vasospasm was markedly more prevalent in the non-viable group (72.7% vs. 9.2%; *p* < 0.001; OR 25.0, 95% CI 5.3–117.8), representing the strongest univariate predictor of failure. A negative O’Brien patency test was also significantly more common in the non-viable group (45.5% vs. 11.5%; *p* = 0.012; OR 0.13). Arterial anastomosis time was significantly prolonged in non-viable flaps (median 45 vs. 25 min; *p* = 0.012; r = 0.256) (Figure 1). A highly significant association was identified between arterial anastomosis time and the occurrence of intraoperative vasospasm: patients who developed vasospasm had markedly prolonged arterial anastomosis times compared with those who did not (median 45 [IQR 35–63] vs. 25 [IQR 19–32] min; *p* < 0.001; r = 0.550). Spearman correlation confirmed this association (rho = 0.352; *p* < 0.001). Stratification by tertile of anastomosis time revealed a dose-dependent gradient in vasospasm prevalence: 9% in the shortest tertile (≤22 min), 0% in the intermediate tertile (22–32 min), and 42% in the longest tertile (>32 min). An anastomosis time exceeding the cohort median (25 min) conferred a more than sixfold increase in the odds of vasospasm (OR 6.43). When the number of anastomosis revisions was analysed as a continuous variable—rather than a binary yes/no indicator—it emerged as a highly significant univariate predictor of failure (median 2 revisions in non-viable vs. 0 in viable flaps; *p* = 0.003; AUC = 0.757), offering greater discriminative power than the dichotomous version of this variable (*p* < 0.001). Spearman correlation analysis revealed strong interdependencies between the number of anastomosis revisions and both total anastomosis time (rho = 0.515; *p* < 0.001) and ischaemia time (rho = 0.520; *p* < 0.001), confirming that these variables represent mechanistically linked facets of intraoperative technical difficulty. Ischaemia time showed a borderline trend towards association with failure (median 110 vs. 55 min in non-viable vs. viable; *p* = 0.061; AUC = 0.674, optimal threshold 110 min) (Figure 2), and ischaemia > 90 min approached significance (54.5% vs. 26.4%; *p* = 0.078). Stratified analysis by flap category demonstrated that this association was driven predominantly by osseous flaps (fibula, iliac crest, and dorsal scapular flaps), in which ischaemia time was significantly longer in the non-viable group (median 95 min (IQR 82–111) viable vs. median 128 min (IQR 115–130) non-viable; *p* = 0.002) (Figure 3), whereas no significant association was observed in fasciocutaneous or myocutaneous flaps. This osseous-flap subgroup finding, based on only 6 failure events among 39 osseous flaps, is exploratory and should be interpreted as preliminary pending confirmation in an independent, adequately powered cohort.

No statistically significant difference in non-viability rate was observed across arterial recipient vessels. The facial artery showed a non-viability rate of 10.6% (7/66; OR 0.80, 95% CI 0.23–2.79; *p* = 0.746), consistent with the overall series rate. The external carotid artery presented the highest non-viability rate among arterial recipients (33.3%; 2/6; OR 4.88, 95% CI 0.91–26.34; *p* = 0.134), though this did not reach statistical significance. The temporal and lingual arteries showed zero failures and zero cases of intraoperative vasospasm (0/7 each).

Regarding venous recipients, anastomosis to the external jugular vein was the only recipient vessel significantly associated with free flap non-viability, with a failure rate of 37.5% (3/8) compared to 8.9% (8/90) for all other venous anastomoses (OR 6.18, 95% CI 1.36–28.09; *p* = 0.044). The thyrolingual trunk (11.9%; OR 1.14, 95% CI 0.34–3.83; *p* = 1.000) and facial vein (9.4%; OR 0.82, 95% CI 0.22–3.06; *p* = 1.000) showed no significant association with flap outcome.

Overall, intraoperative critical vasospasm occurred in 16 of 98 patients (16.3%). When stratified by arterial recipient vessel, the external carotid artery showed a markedly higher vasospasm rate (50.0%; 3/6) compared to all other arterial recipients combined (13/92; 14.1%), with an OR of 5.89 (95% CI 1.20–28.88; *p* = 0.053). Although this finding did not reach conventional statistical significance, the effect size is clinically relevant. No significant difference in vasospasm rates was observed for the facial artery (16.7%; 11/66; OR 1.04, 95% CI 0.34–3.16; *p* = 1.000), thyroid artery (20.0%; 2/10; OR 1.51, 95% CI 0.33–6.90; *p* = 0.665), temporal artery (0/7; *p* = 0.595), or lingual artery (0/7; *p* = 0.595).

The number of red blood cell units transfused within 24 h was also significantly higher in the non-viable group (*p* = 0.033; r = 0.218). Vasopressor use within 24 h (*p* = 0.096) and total anastomosis time (72 vs. 62 min; *p* = 0.100) showed non-significant trends. Derived variables including percentage haemoglobin drop from preoperative to 24-h values (*p* = 0.897; AUC = 0.512) and the hypotension index (cumulative time with MAP < 75 mmHg) did not reach statistical significance, although absolute values were numerically higher in the non-viable group.

### 3.5. Multivariate Logistic Regression

Nineteen variables showed *p* < 0.20 on univariate analysis and were considered for the multivariate model. To limit overfitting given the small non-viable cohort (n = 11), a six-variable model was constructed including the most clinically relevant intraoperative predictors. Several variables that reached statistical significance on univariate analysis (Table 3 and Table 4) did not retain independent significance after multivariable adjustment. Critical vasospasm was the sole independently significant predictor (adjusted OR 0.36; 95% CI 0.16–0.81; *p* = 0.014), indicating a markedly lower probability of viability in patients experiencing vasospasm. Arterial anastomosis time, entered as a standardised (z-score) continuous variable, showed a persistent but non-significant trend (adjusted OR 0.51 per 1-SD [~19-min] increase; 95% CI 0.24–1.08; *p* = 0.079), approximately equivalent to an OR of 0.71 per 10-min increase; this trend indicates a possible but statistically inconclusive independent association that requires confirmation in a larger cohort. The remaining covariates lost significance after adjustment (Table 5).

## 4. Discussion

This prospective cohort study of 98 consecutive oncological head and neck free flap reconstructions identified critical intraoperative vasospasm, arterial anastomosis time, number of anastomosis revisions, as the principal determinants of flap viability on univariate analysis; however, only vasospasm retained independent significance after multivariable adjustment. Preoperative creatinine was the only patient-related variable associated with flap failure. Although low serum creatinine has been proposed as an indirect marker of reduced muscle mass and frailty, creatinine is an imprecise surrogate of sarcopenia, and no established biological mechanism links low creatinine to free flap failure. Therefore, this isolated finding should be interpreted cautiously and may reflect residual confounding or a chance association rather than a true causal relationship. The study confirms the absence of any significant predictive role for a comprehensive preoperative thrombophilia and coagulation panel. These findings are consistent with a growing body of evidence emphasising the primacy of intraoperative technical and vascular factors over preoperative patient characteristics in determining free flap outcomes [1,6].

### 4.1. Overall Flap Survival

The observed free flap survival rate of 88.8% is in the lower range of reports from high-volume centres, which typically report rates of 91–99% [5,6]. This likely reflects the complexity of the case mix: 28 patients (28.6%) had undergone prior head and neck surgery—of whom 14 (14.3%) had a previous microsurgical reconstruction and 11 (11.2%) had experienced prior flap failure—all of which increase operative difficulty, compromise recipient vessel quality, and reduce the availability of suitable anastomotic targets [6]. The relatively higher failure rate may also be attributed to the prospective consecutive nature of the series, which avoids the selection bias inherent to retrospective studies, and to the inclusion of all case complexities without exclusion criteria based on prior oncological treatment.

### 4.2. Arterial Anastomosis Time

The finding that arterial anastomosis time was significantly prolonged in the non-viable group represents one of the central and most novel contributions of this study. This univariate association was accompanied by a consistent, though statistically non-significant, trend on multivariable adjustment (*p* = 0.079), and should accordingly be interpreted as a strong candidate predictor rather than a confirmed independent one. Notably, this variable is rarely reported as a primary outcome predictor in the microsurgical literature. Most published series do not record arterial anastomosis time independently, instead collapsing it into total operative or ischaemia time. Hennocq et al. [1] are, to our knowledge, the only group to have prospectively recorded this variable in a head and neck cohort (mean 29.5 ± 22.1 min across 307 flaps), yet found no significant association with failure in their Cox model (*p* = 0.420). The significant association observed in the present cohort therefore appears to be a genuinely underreported finding, whose detection may depend on case-mix complexity and the proportion of high-risk anastomoses.

Prolonged arterial anastomosis time is best understood as a composite marker of adverse intraoperative conditions: unfavourable vessel anatomy (calcification, size mismatch, short stumps), repeated thrombosis requiring re-anastomosis, and overall technical difficulty. Hillberg et al. [18] explicitly acknowledged this interpretation, noting that “in cases in which ischaemia time might be a reflection of a difficult technical procedure, it remains a predictive indicator for the risk of major complications postoperatively,” while Marre and Hontanilla [17] similarly observed that shorter ischaemia times inherently reflect an easier and more expeditious anastomosis with a lower probability of complications. Each of these adverse conditions independently increases the risk of intimal injury, platelet aggregation, and anastomotic thrombosis, compounded by ischaemia–reperfusion injury as clamping time extends [15].

It must be emphasised, however, that the present study is observational in design and cannot establish causality. It remains equally plausible that arterial anastomosis time functions primarily as a marker of the same underlying technical difficulty—rather than as an independent causal mechanism—that increases the risk of flap failure through unfavourable vessel anatomy, repeated re-anastomosis, or variable surgeon experience; under this interpretation, a longer anastomosis time would be a consequence, rather than a cause, of a technically compromised procedure. Ischaemia–reperfusion injury accumulating during prolonged clamping [15] may still contribute an additional, time-dependent causal component, but the relative contribution of each pathway cannot be disentangled with the present dataset. This distinction has practical implications: while technical training aimed at reducing anastomosis time is likely to be beneficial regardless of the underlying mechanism, it should not be assumed that shortening anastomosis time alone, without addressing the underlying technical difficulty, will proportionally reduce the risk of flap failure.

ROC analysis confirmed that arterial anastomosis time outperformed total ischaemia time as a discriminator of free flap failure (AUC 0.740 vs. 0.674), with the former reaching the threshold for moderate-to-good discriminative capacity (AUC > 0.70). Neither variable achieved perfect discrimination, which is expected given the multifactorial nature of flap failure; however, an AUC of 0.740 for a single intraoperative time variable is clinically meaningful and compares favourably with composite predictive scores reported in other surgical domains. The optimal Youden threshold for total ischaemia time was 110 min—precisely twice the median ischaemia time recorded in viable flaps (55 min) and identical to the median observed in the non-viable group—lending biological plausibility to this cut-off as a practical intraoperative target.

In the multivariate model of the present series, arterial anastomosis time retained a trend towards independent significance (*p* = 0.079) after adjustment for vasospasm and other covariates; however, given the limited statistical power of a cohort with only 11 flap-failure events, this non-significant trend should be interpreted cautiously as hypothesis-generating rather than confirmatory evidence of a genuine independent biological effect. Critically, when the number of anastomosis revisions was modelled as a continuous variable, it emerged as one of the strongest univariate predictors of failure (*p* = 0.003), with a median of two revisions in failed flaps versus none in viable flaps—directly corroborated by Hennocq et al. [1], who identified per- and postoperative anastomosis revision as an independent predictor of failure on multivariate Cox analysis (*p* = 0.041) in their prospective head and neck cohort. Spearman correlation analysis further confirmed the mechanistic coherence of these findings: revision count correlated strongly with both total anastomosis time (*p* < 0.001) and ischaemia time (*p* < 0.001), underscoring that each revision simultaneously adds to cumulative ischaemic injury and signals vessel-level technical adversity. It should be acknowledged, however, that the prolonged anastomosis times and higher revision rates observed in failed cases may partly reflect the teaching nature of our unit, where supervised trainees at varying stages of microsurgical proficiency routinely participate in anastomosis performance. In this context, anastomosis time may capture not only intrinsic vessel-level difficulty but also surgeon experience and training level—a confounding factor that prospective studies specifically designed to disentangle technical and educational variables would be required to address. These data support a specific focus on arterial anastomosis efficiency in surgical training programmes, and suggest that the number of revisions may serve as a real-time intraoperative prognostic indicator warranting heightened postoperative surveillance, although this observation remains exploratory and requires prospective validation before implementation.

### 4.3. Intraoperative Vasospasm

Vasospasm emerged as the strongest predictor of flap failure in this series, both in univariate (*p* < 0.001) and multivariate analyses (*p* = 0.014). Importantly, the vasospasm recorded in our series corresponds specifically to critical intraoperative vasospasm, defined as complete cessation of arterial blood flow requiring pharmacological or mechanical intervention—a more stringent criteria than simple pedicle spasm. Contrary to the commonly held assumption that vasospasm preferentially affects small-calibre terminal recipient vessels, stratification by arterial recipient revealed no excess vasospasm rate at the facial artery, thyroid artery, temporal artery, or lingual artery. Instead, the external carotid artery exhibited a markedly higher vasospasm rate (*p* = 0.053), suggesting that vessel anatomy, surgical context, and proximity to autonomic neural structures can be more determinant of vasospasm risk than vessel calibre alone.

Vasospasm in the microsurgical context is a reflex smooth muscle contractile response to mechanical stimulation, thermal stress, or chemical mediators, affecting both pedicle and recipient vessels. The higher susceptibility of the external carotid artery may reflect its proximity to the sympathetic fibres of the carotid sheath, rendering it more reactive to adrenergic vasoconstrictive stimuli during dissection [21,22]. However, an important selection bias must be acknowledged: in our unit, anastomosis to the external carotid artery is typically reserved for cases in which preferred recipient vessels—the facial artery, thyrolingual trunk, or facial vein—are unavailable due to prior surgery, radiation-induced fibrosis, or anatomical loss. These are inherently the most complex cases in the series, with the most compromised operative fields and the greatest baseline vascular adversity. The higher vasospasm and failure rates observed at the external carotid artery may therefore partly reflect this underlying case complexity rather than a vessel-specific susceptibility, representing a confounding effect that the current analysis is underpowered to fully disentangle. This may also explain why our finding diverges from reports in the broader microsurgical literature, where recipient vessel selection tends to reflect surgeon preference rather than anatomical necessity. Pharmacological management remains empirical and heterogeneous across centres: Vargas et al. performed a systematic review of 20 studies evaluating 14 vasodilatory agents and found no single agent demonstrating clear superiority [22], while Hyza et al. demonstrated in an experimental rat model that 10% topical magnesium sulphate was the most effective agent for reducing vasospasm duration, whereas topical 2% lidocaine paradoxically prolonged it [21]—findings with direct translational implications, particularly when the external carotid artery is selected as recipient.

A statistically significant and clinically substantial association was identified between arterial anastomosis time and intraoperative vasospasm. These findings suggest that arterial anastomosis time and vasospasm do not represent independent risk pathways but are mechanistically linked: prolonged microvascular manipulation—through repetitive endothelial trauma, sustained mechanical distension of the vessel wall, topical antispasmodic agent depletion, and cumulative smooth muscle stimulation—plausibly triggers the reflexive vasoconstrictive response that constitutes vasospasm. Within the subgroup of patients who did develop vasospasm, arterial anastomosis time remained numerically longer in those whose flaps ultimately failed compared with those in whom salvage was successful (median 54 vs. 38 min; *p* = 0.052), suggesting that anastomosis duration may modulate outcome even after vasospasm has been established. Taken together, these data are consistent with a sequential mechanistic cascade in which a technically demanding or complicated arterial anastomosis precipitates vasospasm, which in turn propagates ischaemic injury and anastomotic thrombosis. This cascade model implies that arterial anastomosis time is not merely a surrogate for technical difficulty but may function as a modifiable upstream determinant of vasospasm, elevating its prognostic relevance beyond what its independent OR in the multivariate model alone would suggest.

### 4.4. Ischaemia Time

Although neither total ischaemia time nor ischaemia > 90 min reached statistical significance in this cohort (*p* = 0.061 and *p* = 0.078, respectively), the effect sizes observed and their consistent direction are noteworthy.

Stratification by flap tissue category revealed that this global borderline result masks a clinically important but exploratory and statistically robust effect in osseous flaps (fibula, scapular and iliac crest; n = 39): ischaemia time was significantly prolonged in non-viable compared to viable osseous flaps (*p* = 0.002), whereas no significant association was observed in fasciocutaneous (n = 42) or myocutaneous flaps (n = 17, no failures). This differential effect is biologically plausible: osseous and osteomyocutaneous flaps contain cortical bone and periosteal tissue with substantially lower ischaemic tolerance than cutaneous or muscular components. Our series comprised 30 fibula osteomyocutaneous flaps—the same flap type examined by Chang et al. [3], who reported in 116 consecutive head and neck fibula reconstructions that ischaemia below five hours did not increase complications, whereas times exceeding this threshold did (*p* = 0.03). The substantially lower threshold emerging from our osseous subgroup (110–128 min vs. 300 min) likely reflects differences in primary endpoint: Chang et al. captured any postoperative complication, whereas our endpoint was complete flap non-viability—a more severe event requiring earlier and more critical vascular compromise. Taken together, both datasets converge on the message that ischaemia duration is a meaningful biological variable in fibula flaps, with its effect apparent at lower thresholds when the endpoint is flap loss. In contrast, Politano et al. [20], in a systematic review and meta-analysis of head and neck microvascular free tissue transfer, found no significant association between ischaemia time and acute complications across flap types (*p* = 0.98), a result likely attributable to the dilutional effect of tissue types with higher ischaemic tolerance within a heterogeneous dataset.

The detrimental effect of prolonged ischaemia has been more consistently demonstrated in non-head-and-neck contexts. Ehrl et al. found significantly higher rates of complications and revision surgery for ischaemia ≥ 60 min across 690 free flap reconstructions of various types (*p* < 0.05) [16]. Marre and Hontanilla identified ischaemia time as an independent risk factor for microvascular complications in DIEP flap breast reconstruction (OR 3.81, *p* = 0.03), with risk increasing beyond 90–120 min [17]. Hillberg et al. similarly demonstrated in 677 DIEP flaps that ischaemia ≥ 60 min was independently associated with major complications (*p* = 0.016) [18]. Arellano et al., in a meta-analysis of 17 studies and 6884 breast free flaps, confirmed that prolonged ischaemia increases postoperative complication risk, with venous events predominating in failure cases [19].

The mechanistic basis is well established: Stephan et al. demonstrated in a porcine free flap model that transfer-related ischaemia produces a continuous pH drop, electrolyte disturbances, and haematological changes in the flap venous blood [15], while Bhullar et al. highlighted the role of perfusion-related anatomical factors—perforator number, location, and venous system anatomy—in determining flap tolerance to ischaemia [23]. The absence of a statistically significant global finding in our series most likely reflects limited statistical power given the small non-viable cohort, rather than a true absence of effect.

### 4.5. Recipient Vessel Selection, Venous Anastomosis, and Haemodynamic Factors

The identification of external jugular vein anastomosis as a significant univariate risk factor for free flap failure represents one of the clinically actionable findings of this series and is consistent with a growing body of evidence emphasising the primacy of venous recipient selection in determining flap outcome. Hennocq et al. [1], in a prospective cohort of 307 consecutive head and neck free flaps, identified anastomosis to the anterior jugular vein and superior thyroid vein as independent risk factors for failure on multivariate analysis, explicitly concluding that venous anastomosis choice exerts greater influence on outcome than patient-level characteristics. Las et al. [6] similarly reported that anastomosis to the lingual vein or superficial temporal artery were independently associated with failure in a retrospective cohort of 1530 free flaps. Kubo et al. [24] further demonstrated that venous obstruction is more common than arterial obstruction in head and neck microsurgery, and that anatomical factors at the venous anastomosis—vessel calibre, flow dynamics, and tethering—are critical determinants of thrombotic risk. Taken together with our data, these findings consistently underscore that venous recipient selection is a modifiable surgical variable with direct consequences for flap survival.

The higher failure rate associated with external jugular vein anastomosis in our series likely reflects its specific anatomical disadvantages: a superficial course susceptible to kinking and extrinsic compression during neck positioning and wound closure, greater positional variability compared to the thyrolingual trunk or facial vein, and a tendency towards calibre mismatch with flap pedicle veins that predisposes to anastomotic turbulence and thrombus formation. These findings support a preference for the thyrolingual trunk or facial vein as venous recipients when anatomically feasible, reserving the external jugular vein for cases in which no suitable alternative exists. It should be noted, however, that this association is based on only eight patients undergoing external jugular vein anastomosis, of whom three experienced flap loss, and the wide 95% confidence interval (1.36–28.09) reflects the statistical instability of this estimate; prospective validation in larger series is required before definitive clinical guidance can be established.

### 4.6. O’Brien Patency Test

The significantly higher rate of negative O’Brien test in the non-viable group (*p* = 0.012) confirms the intraoperative utility of this simple and reproducible manoeuvre for the early detection of anastomotic compromise. The O’Brien test—performed by gently occluding the vessel distal to the anastomosis and observing refilling after release—provides real-time qualitative information about blood flow and anastomotic patency and was applied routinely in our unit at the completion of every arterial anastomosis. Crucially, the negative results recorded in our series do not reflect unresolved anastomotic compromise at the time of wound closure: in all cases, the anastomosis was revised until a positive patency test was achieved before the procedure was concluded. Rather, a negative O’Brien test at any point during the operation—even when subsequently corrected—was associated with a significantly higher rate of flap loss during the inpatient period. This finding reframes the O’Brien test not merely as a binary indicator of immediate patency, but as an intraoperative marker of vascular adversity: a vessel that requires revision to achieve flow is one that has already been subjected to greater mechanical stress, intimal manipulation, and cumulative ischaemic injury, all of which predispose to late thrombosis even after apparent restoration of patency. These data therefore support heightened postoperative surveillance in any patient who required anastomotic revision to achieve a positive patency test, regardless of the final intraoperative status.

### 4.7. Haemodynamic Factors

Vasopressor use within 24 h showed a trend towards association with flap failure in our series (*p* = 0.096), consistent with the concern that vasoconstrictive agents may compromise peripheral flap perfusion during the critical postoperative window [25]. Although definitive evidence on this relationship remains limited, the available data suggest that targeted haemodynamic management—maintaining adequate mean arterial pressure while minimising vasopressor dependence—represents the most rational approach to postoperative care in microsurgical patients, and warrants prospective evaluation in dedicated studies.

### 4.8. Limitations

Although this prospective study represents a considerable series of consecutive reconstructive free flap procedures performed at a single tertiary referral centre, providing robust real-world data on flap viability and its determinants, several limitations should be acknowledged. First, the non-viable cohort (n = 11) is small, limiting statistical power and the reliability of multivariate modelling. Specifically, the multivariate model included six variables for 11 failure events, yielding an events-per-variable ratio (EPV) of approximately 1.8, well below the recommended minimum of 10; although L2 regularisation was applied to mitigate overfitting, the resulting coefficient estimates and confidence intervals should be interpreted accordingly. This limited number of events also means that the exploratory time thresholds proposed in this study (45 min for arterial anastomosis time; 110 min for total ischaemia time; the osseous-flap ischaemia threshold) and the osseous-flap subgroup findings, although internally consistent and biologically plausible, are derived from very small numbers of events and must be regarded as preliminary and hypothesis-generating rather than validated clinical cut-offs; external validation in an independent, adequately powered cohort is required before their incorporation into clinical protocols. The relatively small total sample size (n = 98) necessitates caution in interpreting trends that approach but do not reach conventional significance. Second, while data were collected prospectively, detailed intraoperative anatomical characterisation of recipient vessels—such as vessel diameter, wall quality, and degree of atherosclerosis—was not systematically recorded, precluding subgroup analyses by vessel characteristics. Third, the single-centre design limits external generalisability, and temporal changes in surgical team composition, learning curves, and anaesthetic protocols may introduce some heterogeneity over the four-year study period. Fourth, the multivariate model employed a ridge penalty to mitigate overfitting, which may introduce coefficient bias; Firth’s penalised likelihood regression would be preferred in future studies with such low event rates. Finally, follow-up beyond hospital discharge was not systematically captured in this analysis; delayed vascular complications occurring after discharge are therefore not reflected in the outcome variable.

## 5. Conclusions

In this prospective cohort of 98 consecutive oncological head and neck microsurgical reconstructions, arterial anastomosis time, number of anastomosis revisions, and intraoperative vasospasm—which were found to be significantly and dose-dependently interrelated—were identified as the principal univariate predictors of free flap failure. Vasospasm was the sole independently significant variable in multivariate analysis (*p* = 0.014), with arterial anastomosis time showing a persistent and clinically relevant trend (*p* = 0.079). In contrast, preoperative epidemiological factors, comorbidities, and a comprehensive coagulation and thrombophilia panel showed no significant association with flap viability.

These findings highlight the primacy of intraoperative technical and vascular factors over preoperative patient characteristics as determinants of free flap outcome in this context. Clinically, they support the prioritisation of anastomotic technical training, early recognition and treatment of intraoperative vasospasm, systematic use of the O’Brien patency test before wound closure, and judicious management of ischaemia time. Prospective multicentre studies with larger non-viable cohorts are needed to confirm these associations, develop validated predictive tools incorporating intraoperative variables, and establish evidence-based protocols for vasospasm management and anastomotic decision-making. On the basis of the present findings, and acknowledging the exploratory nature of these estimates given the small number of flap failures (n = 11), we propose that an arterial anastomosis time exceeding the Youden-optimal threshold of 45 min and the occurrence of intraoperative critical vasospasm may serve as candidate real-time intraoperative warning triggers for heightened postoperative monitoring, pending prospective validation of these thresholds in an independent cohort before their adoption into clinical protocols.

## Figures and Tables

**Table 1:** Baseline patient characteristics.

Characteristic	Total (N = 98) n (%)	Median [IQR]/n (%)
Age (years)	N = 98	64 [54–74]; mean 62.9 ± 14.4
Female sex	42/98	42.9%
Male sex	56/98	57.1%
Smoking habit	43/98	43.9%
Alcohol consumption	20/97	20.6%
Hypertension	43/98	43.9%
Dyslipidaemia	29/98	29.6%
Diabetes mellitus	12/98	12.2%
Ischaemic heart disease	3/98	3.1%
Peripheral arterial disease	1/98	1.0%
Personal history of DVT/PE	7/98	7.1%
Chronic liver disease	5/98	5.1%
Chronic kidney disease	1/98	1.0%
BMI > 30 kg/m^2^	6/98	6.1%
Anticoagulant therapy	11/98	11.2%
Antiplatelet therapy	5/98	5.1%
Reduced mobility	5/98	5.1%
Active oncological process	71/98	72.4%
Primary reconstruction	82/98	83.7%
Previous microsurgical reconstruction	14/98	14.3%
Previous flap failure	11/98	11.2%
Prior radiotherapy	24/98	24.5%

DVT: deep vein thrombosis; PE: pulmonary embolism; BMI: body mass index.

**Table 2:** Flap and surgical characteristics.

Surgical Variable	N	n (%) or Median [IQR]
Flap type		
Fibula osteomyocutaneous flap	30/98	30.6%
Radial forearm fasciocutaneous flap	24/98	24.5%
Anterolateral thigh fasciocutaneous flap	18/98	18.4%
Vastus lateralis myocutaneous flap	14/98	14.3%
Iliac crest osteomuscular flap	6/98	6.1%
Transverse rectus abdominis myocutaneous flap	3/98	3.1%
Latissimus dorsi-scapular osteomyocutaneous flap	3/98	3.1%
Anastomosis		
Main arterial recipient—facial artery	66/98	67.3%
Main venous recipient—thyrolingual trunk	42/98	42.9%
Surgery duration (min)	98	470 [390–602]
Total ischaemia time (min)	98	55.5 [35–95]
Ischaemia time > 90 min	29/98	29.6%
Arterial anastomosis time (min)	98	25 [19–35]
Venous anastomosis time (min)	98	35 [29–44]
Total anastomosis time (min)	98	64 [51–75]
Positive O’Brien patency test	83/98	84.7%
Intraoperative critical vasospasm	16/98	16.3%
MAP < 75 mmHg in OR (min)	98	45 [15–95]
MAP < 75 mmHg in ICU (min)	98	75 [30–195]
Total MAP < 75 mmHg first 24 h (min)	98	108 [75–318]
Total MAP < 75 mmHg first 24 h (%)	98	7.5% [5.2–22.0]
RBC transfusion within 24 h	70/98	71.4%
Vasopressor use within 24 h	66/98	67.3%

MAP: mean arterial pressure; OR: operating room; ICU: intensive care unit; RBC: red blood cells.

**Table 3:** Univariate comparative analysis of categorical variables.

Variable	Viable (n = 87) n (%)	Non-Viable (n = 11) n (%)	*p*-Value *	OR (95% CI)
Female sex	35/87 (40.2%)	7/11 (63.6%)	0.197	2.58 (0.68–9.83)
Smoking habit	38/87 (43.7%)	5/11 (45.5%)	1.000	1.07 (0.30–3.82)
Alcohol consumption	17/86 (19.8%)	3/11 (27.3%)	0.692	1.52 (0.37–6.25)
Hypertension	38/87 (43.7%)	5/11 (45.5%)	1.000	1.07 (0.30–3.82)
Dyslipidaemia	26/87 (29.9%)	3/11 (27.3%)	1.000	0.88 (0.22–3.57)
Diabetes mellitus	12/87 (13.8%)	0/11 (0.0%)	0.350	—
Personal history of DVT/PE	6/87 (6.9%)	1/11 (9.1%)	0.578	1.35 (0.14–12.5)
Anticoagulant therapy	9/87 (10.3%)	2/11 (18.2%)	0.607	1.93 (0.36–10.4)
Active oncological process	63/87 (72.4%)	8/11 (72.7%)	1.000	1.02 (0.26–3.95)
Prior radiotherapy	21/87 (24.1%)	3/11 (27.3%)	1.000	1.18 (0.29–4.83)
Previous microsurgical reconstruction	11/87 (12.6%)	3/11 (27.3%)	0.190	2.62 (0.61–11.3)
Previous flap failure	8/87 (9.2%)	3/11 (27.3%)	0.106	3.73 (0.84–16.6)
Ischaemia time > 90 min	23/87 (26.4%)	6/11 (54.5%)	0.078	3.31 (0.88–12.5)
Positive O’Brien patency test	77/87 (88.5%)	6/11 (54.5%)	0.012 †	0.13 (0.03–0.58)
Intraoperative critical vasospasm	8/87 (9.2%)	8/11 (72.7%)	<0.001 ‡	25.0 (5.3–117.8)
RBC transfusion within 24 h	61/87 (70.1%)	9/11 (81.8%)	0.724	1.87 (0.38–9.21)
Vasopressor use within 24 h	56/87 (64.4%)	10/11 (90.9%)	0.096	5.18 (0.62–43.1)

* Fisher’s exact test; OR: odds ratio; CI: confidence interval; DVT: deep vein thrombosis; RBC: red blood cells. † *p* < 0.05; ‡ *p* < 0.001.

**Table 4:** Univariate comparative analysis of continuous variables (Mann–Whitney U test).

Variable	Viable (n = 87) Median [IQR]	Non-Viable (n = 11) Median [IQR]	*p* *	r
Age (years)	64 [54–74]	71 [57–75]	0.562	0.063
Haemoglobin (g/dL)	14.3 [13.1–15.0]	13.5 [10.2–14.7]	0.147	0.149
Haematocrit (%)	42.1 [39.0–44.9]	40.7 [29.4–45.0]	0.347	0.096
Platelets (×10^3^/µL)	243 [198–303]	240 [212–264]	0.723	0.037
Leucocytes (×10^3^/µL)	7.75 [6.11–9.73]	6.38 [4.60–10.0]	0.350	0.095
Creatinine (mg/dL)	0.88 [0.72–1.02]	0.65 [0.61–0.79]	0.009	0.263
GOT (U/L)	20 [16–25]	20 [17–29]	0.809	0.025
Fibrinogen (mg/dL)	407 [350–509]	441 [353–596]	0.317	0.102
Prothrombin activity (%)	96.7 [87.8–106]	87.8 [81.0–97.8]	0.151	0.146
aPTT (s)	29.3 [27.6–32.6]	32.0 [28.7–33.7]	0.392	0.086
D-dimer (µg/L)	410 [234–561]	395 [329–635]	0.585	0.057
Protein C (%)	109.5 [92.3–132.9]	95.0 [90.5–125.0]	0.520	0.067
Protein S (%)	100.5 [88.2–115.3]	101.0 [79.6–102.9]	0.248	0.116
Antithrombin (%)	103 [89.0–115]	114.7 [101.3–128.8]	0.063	0.186
ACA IgG	3.03 [1.70–5.36]	3.35 [1.68–4.88]	0.783	0.028
Anti-β2GP IgG	2.33 [1.05–4.25]	2.50 [1.07–4.12]	0.937	0.008
Padua score	3 [3–4]	3 [3–4]	0.813	0.024
IMPROVE score	3 [3–4]	4 [3–5]	0.122	0.157
Surgery duration (min)	466 [390–595]	555 [413–622]	0.362	0.092
Ischaemia time (min)	55 [35–94]	110 [41–128]	0.061	0.190
Arterial anastomosis time (min)	25 [19–35]	45 [29–62]	0.012	0.256
Venous anastomosis time (min)	35 [29–43]	34 [24–44]	0.574	0.059
Total anastomosis time (min)	62 [51–75]	72 [64–117]	0.100	0.168
MAP < 75 first 24 h (min)	105 [75–235]	465 [75–778]	0.180	0.134
No. of RBC units transfused	2 [1–2]	2 [2–2]	0.033	0.218

* *p*-value (Mann–Whitney U); r: rank-biserial correlation coefficient (effect size). aPTT: activated partial thromboplastin time; ACA: anticardiolipin antibody; MAP: mean arterial pressure. β2GP: beta2-glycoprotein.

**Table 5:** Multivariate logistic regression analysis for predictors of free flap non-viability.

Variable	Adj. OR	95% CI	*p*-Value
Intraoperative critical vasospasm	0.36	0.16–0.81	0.014 †
Arterial anastomosis time (min)	0.51	0.24–1.08	0.079
Negative O’Brien patency test	0.87	0.42–1.84	0.723
Ischaemia time > 90 min	1.66	0.58–4.71	0.341
Creatinine (mg/dL)	3.82	0.54–27.2	0.180
Vasopressor use within 24 h	0.71	0.22–2.27	0.561

Adj. OR: adjusted odds ratio (outcome = viability; OR < 1 indicates association with non-viability). L2-penalised logistic regression (λ = 0.1). † *p* < 0.05. Continuous variables (arterial anastomosis time, creatinine) were standardised (z-scored; mean = 0, SD = 1) before entry into the model.

## Data Availability

The data presented in this study are available on request from the corresponding author due to patient privacy considerations.
